# LPEseq: Local-Pooled-Error Test for RNA Sequencing Experiments with a Small Number of Replicates

**DOI:** 10.1371/journal.pone.0159182

**Published:** 2016-08-17

**Authors:** Jungsoo Gim, Sungho Won, Taesung Park

**Affiliations:** 1 Institute of Health and Environment, Seoul National University, 1 Gwanak-ro, Gwanak-gu, Seoul, Korea; 2 Graduate School of Public Health, Seoul National University, 1 Gwanak-ro, Gwanak-gu, Seoul, Korea; 3 Department of Statistics, Seoul National University, 1 Gwanak-ro, Gwanak-gu, Seoul, Korea; Queen's University Belfast, UNITED KINGDOM

## Abstract

RNA-Sequencing (RNA-Seq) provides valuable information for characterizing the molecular nature of the cells, in particular, identification of differentially expressed transcripts on a genome-wide scale. Unfortunately, cost and limited specimen availability often lead to studies with small sample sizes, and hypothesis testing on differential expression between classes with a small number of samples is generally limited. The problem is especially challenging when only one sample per each class exists. In this case, only a few methods among many that have been developed are applicable for identifying differentially expressed transcripts. Thus, the aim of this study was to develop a method able to accurately test differential expression with a limited number of samples, in particular non-replicated samples. We propose a local-pooled-error method for RNA-Seq data (LPEseq) to account for non-replicated samples in the analysis of differential expression. Our LPEseq method extends the existing LPE method, which was proposed for microarray data, to allow examination of non-replicated RNA-Seq experiments. We demonstrated the validity of the LPEseq method using both real and simulated datasets. By comparing the results obtained using the LPEseq method with those obtained from other methods, we found that the LPEseq method outperformed the others for non-replicated datasets, and showed a similar performance with replicated samples; LPEseq consistently showed high true discovery rate while not increasing the rate of false positives regardless of the number of samples. Our proposed LPEseq method can be effectively used to conduct differential expression analysis as a preliminary design step or for investigation of a rare specimen, for which a limited number of samples is available.

## Introduction

High-throughput sequencing of cDNA derived from an RNA sample, known as RNA-Seq, has recently been developed and applied to various studies depending on the scientific interests such as detecting fusion genes, transcribed single nucleotide polymorphisms (SNPs), and differential expression (hereafter, DE) [1, 2]. In particular, profiling gene expression and testing DE between classes have been the primary process of many biological studies.

The main purpose of DE analysis is to identify transcripts that changed significantly in abundance across experimental conditions. This goal has been achieved using different statistical methods for data from array-based technologies. Compared to microarray, however, RNA-Seq has different characteristics such as high dynamic range and low background expression level. In order to address those properties, various methods have been proposed using Poisson and negative binomial distributions [3–5].

The software edgeR assumes that the mean and variance are related with a single proportionality constant that is the same throughout the experiments; thus, only one parameter needs to be estimated for each transcript [4]. Instead, DESeq decomposes the variance into two terms, a shot-noise term and a raw-variance term [5]. By assuming that the raw variance of each transcript is a function of the expectation value of a transcript’s concentration and condition, DESeq extends the model proposed by edgeR. NBPSeq uses an over-parameterized version of the negative binomial called “NBP distribution”, which incorporates a non-constant dispersion parameter directly within a parametric family [6]. All these three methods are based on similar principles, i.e., explicit modeling of the counts using a negative binomial distribution.

Comprehensive reviews on pre-existing methods for DE analysis with both real and simulation datasets has been recently published. By examining 11 methods including edgeR [4], DESeq [5], and NBPSeq [6], it was reported that all methods performed well with large sample sizes, while most of them showed weakness in DE analysis with small sample sizes [7]. A similar result was obtained in Seyednasrollah et al’s paper; unlike the study with large sample size, the choice of the method becomes critical when the number of samples is small [8]. With small sample size (two or three samples) per each class, the review papers reported that the methods based on negative binomial modelling, such as edgeR and DESeq, displayed relatively better performance [7–9].

Despite the decreasing sequencing costs, RNA-Seq experiments remain expensive to allow extensive biological replications. Moreover, limited specimen availability often leads to studies with a small number of (sometimes none of) replicates. Unfortunately, many of the existing methods have been largely unsuccessful in DE analysis with a small number of samples, and few researches have correctly addressed the problem arisen from non-replicated samples per each class. Therefore, there remains the need for an effective method that could be applicable to DE analysis with small samples or with one sample per each class.

Here, we proposed a method, named local-pooled-error with RNA-Sequencing data (LPEseq), which proposes to analyze DE detection of RNA-Seq experiments with a small number of or non-replicated samples in each class. Based on the LPE method [10], we extended the protocol to RNA-Seq data with a non-replicated set by introducing additional processes. Then, we compared the results obtained from the proposed method with those from several other techniques using both real and simulated datasets. Because it was reported that the performance of edgeR, DESeq, and NBPSeq with a small number of replicates is slightly better than that of other methods [7], these methods were chosen to compare the results with those obtained using our proposed method for replicated data analysis. Since DESeq was updated to DESeq2 recently, DESeq2 was also included in comparison [11]. However, for non-replicated data sets, only edgeR and DESeq are applicable and, thus, the comparison was made among edgeR, DESeq, and our proposed method for non-replicated data analysis. We found that LPEseq generally shows similar performance to other methods in the presence of replicates but performs better for non-replicated data sets.

## Materials and Methods

### A brief review of the LPE method

Jain *et al*. developed the original LPE method, which pools the error in each local intensity bin and shrinks each error variance estimate toward the mean of other probes (or genes) with similar intensities, for microarray experiments in which gene expression intensity is continuous [10]. The method first evaluates the baseline error distribution for each of the compared experimental conditions, say class *X* and *Y*, respectively. For duplicated arrays (subscript with 1 or 2) in each class, for instance in class *X*, the mean $\left(A=\frac{x_{1}+x_{2}}{2}\right)$ and difference (*M* = ±|*x*_1_−*x*_2_|) values between arrays are evaluated first. Then the variance of *M* on predetermined quantiles of *A* is evaluated and a cubic smoothing spline is fit to the variance estimates on the quantiles. The baseline error distribution for class *Y* is derived using the same way. The test statistic of the LPE method is calculated as follows:

$$Z=\frac{Med(X)-Med(Y)}{{\hat{\sigma }}_{pooled}^{2}}$$ (1)

where Med(.) denotes median and,

$${\hat{\sigma }}_{pooled}^{2}=\tau \left(\frac{{\hat{\sigma }}_{X}^{2}}{n_{X}}+\frac{{\hat{\sigma }}_{Y}^{2}}{n_{Y}}\right),$$ (2)

*n*_*X*_ and *n*_*Y*_ are the number of replicates in the two array samples being compared; ${\hat{\sigma }}_{X}^{2}$ and ${\hat{\sigma }}_{Y}^{2}$ are the estimates of variance of the genes in class *X* and *Y*, respectively; and *τ* is a scaling factor [10].

The LPE method assumes that genes with similar observed intensities have similar expression variances. Based on this assumption, it estimates the gene-specific variance from a calibration curve derived from pooling the variance estimates of replicated expression differences of genes within similar expression intensities. Since the method is based on calculating the error variance from replicated experiments in the same class, it cannot be directly applied to experiments with no biological replicates in one or both classes. Furthermore, the method is suited for the analysis of continuous probe intensities on microarray platforms, unlike the read counts measured on sequencing technology.

### The proposed method: LPEseq

In this study, we improved the LPE method by focusing on two refinement aspects: applicability both to count measurement in RNA-Seq and to experiments with no replicates in each class. We describe here how we addressed these two aspects into the model. Simply, for the former case, the count variable is treated as a continuous variable by performing normalization and log-transformation. For the latter case, we treat two non-replicated samples as if they were replicates and remove the outliers so to reduce the impact on the LPE estimation. For RNA-Seq experiments with replicates, the proposed method conducts the similar procedure, *i*.*e*., estimating the variance of *M* from replicates in each class *X* and *Y*, as was described in the previous section.

To be more specific, we focused our attention on the problem of inferring DE between two different classes. Let $x_{ij}^{\prime }$ and $y_{ij}^{\prime }$ represent the number of read counts mapped to a specific transcript (or gene) *i* in the *j*_*th*_ sample (or replicate) from the experimental condition or class *X* and *Y*, respectively. Since $x_{ij}^{\prime }$ and $y_{ij}^{\prime }$ are influenced by the sequencing depth, these values are not directly comparable. Instead, the relative abundance of the transcripts across the samples can be normalized. Each transcript’s read count was divided by the total number of read counts for that experiment and was log-transformed, i.e., $x_{ij}=\log_{2}\left(x_{ij}^{\prime }/\sum_{i}x_{ij}^{\prime }\right)$ and $y_{ij}=\log_{2}\left(y_{ij}^{\prime }/\sum_{i}y_{ij}^{\prime }\right)$.

Without replicates, only a single measurement in each class is available; thus, the subscript *j* can be dropped without ambiguity. Let *x*_*i*_ and *y*_*i*_ denote the normalized log-transformed count values of the transcript *i* under two different classes *X* and *Y*, respectively. The baseline error distribution was obtained by regarding each sample in different classes as replicates. Under this additional assumption, the *M* and *A* values were evaluated using two samples (each from different classes)., i.e., *M* = ±|*y*−*x*| and $A=\frac{x+y}{2}$. Then $\sigma_{M_{\left(k\right)}}^{2}$, the variance of *M* of the genes on the predetermined *k*_*th*_ bin of *A*, was calculated in the same manner as for replicate analysis. In this case, however, since the variance of *M* is not drawn from the same class, differentially abundant transcripts could act as outliers and adversely affect proper evaluation of the LPE per each class.

**Fig 1:**
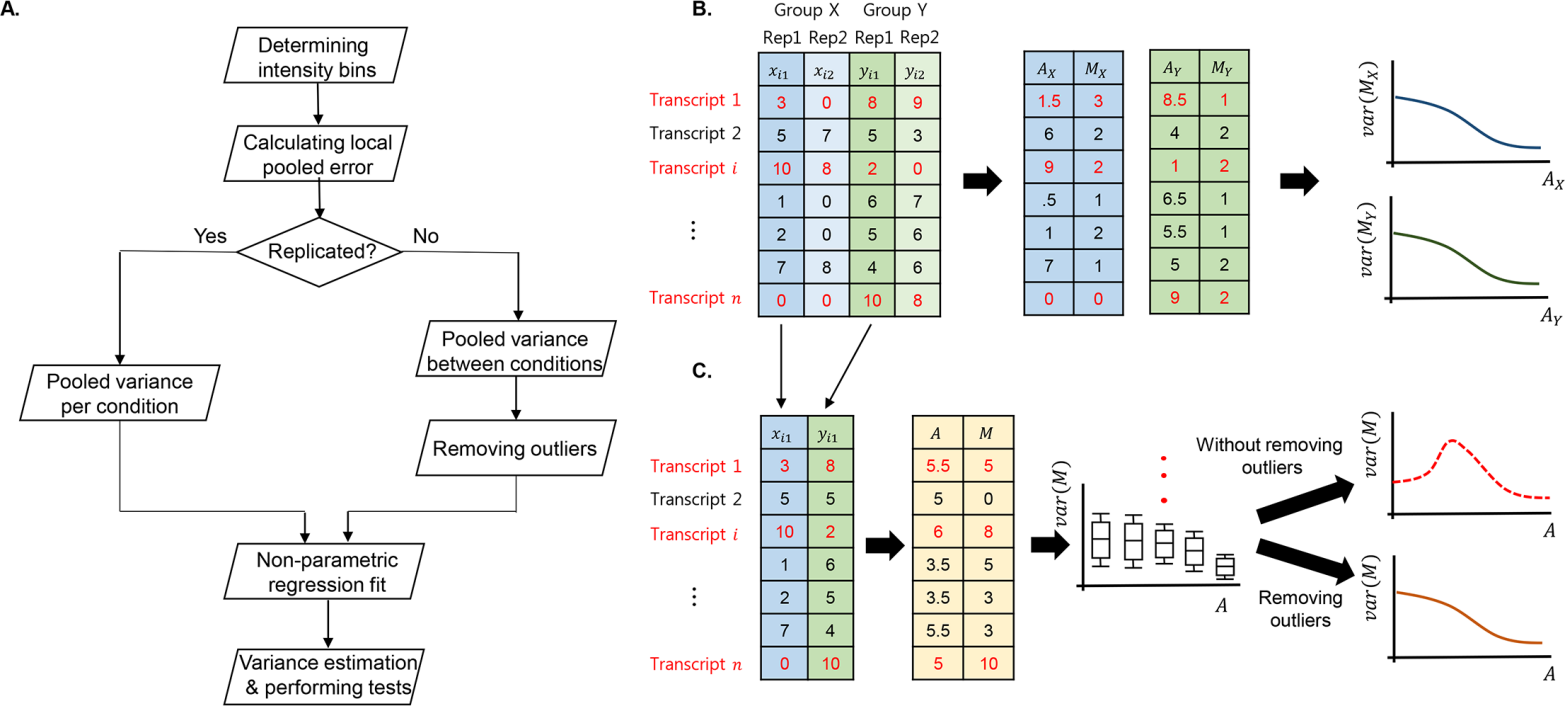
Schematic representation of the local-pooled-error method for RNA-Seq data (LPEseq) method. (A) The flow chart of the proposed algorithm. The proposed method first determines intensity bins (percentile by default) and evaluates the LPE distribution differently depending on the existence of replicates in each class: LPE per each class with replicates and LPE between classes with non-replicated experiments. For non-replicated cases, the addition step smoothens the LPE distribution by removing outliers. Detailed examples are depicted in case of replicated (B) and non-replicated (C) experiments. Blue and green colors represent different classes (i.e., X and Y). The red dotted line and orange line represent the LPE curve with and without outliers, respectively. DE transcripts are colored in red.

Here, we give an example to understand the problem. Suppose we have a dataset consists of *n* transcripts duplicated in two different groups, say X and Y. Three transcripts among *n* transcripts are differentially expressed as shown in Fig 1B and 1C (DE transcripts are colored in red in the figure). With replicate information, the LPEs are drawn from each class separately as described the previous section (Fig 1B), whereas those with non-replicated experiments have to be evaluated using two single observations from two different classes (Fig 1C). When evaluated with non-replicated data, the LPEs of the three DE transcripts (colored in red in the Fig 1B and 1C) show a clear distinction compared to those with replicates and, thus, the fitted variance curve might be biased (red dashed curve in Fig 1C) due to these apparent outliers. By denoting an outlier in each quantile of a given data, we can remove outlying observations and recalibrate an LPE curve less affected by outliers (orange solid curve in Fig 1C).

For removing outliers, both formal (or tests of discordancy) tests and informal (outlier labeling) methods can be used. Formal tests based on *Z*-score [($Z_{i}=\frac{x_{i}-\bar{x}}{sd}$, where *x*_*i*_ ∼ *N*(*μ*,*σ*^2^), where *sd* denotes the standard deviation of data] usually assume a well-behaving distribution, and test if the target extreme observation is an outlier of that distribution. It is obvious that these tests largely depend on the type of observations and distribution assumed [12]. On the other hand, informal methods usually generate an interval or criterion for outlier detection instead of hypothesis testing. Therefore, any observations beyond this criterion could be considered as an outlier. LPEseq provides various strategies for detecting outliers and the criterion of fold difference between classes was used as the default one.

We summarize the detailed procedure of the LPE analysis without replicates as follows:

1. Calculate the mean intensity of transcript *i* in two conditions [i.e., (*x*_*i*_ + *y*_*i*_)/2].
2. Calculate quantiles (percentiles by default) with the mean intensities of the whole transcripts evaluated at step I, and define “intensity bins” using adjacent quantile values. Thus the width of the bin depends on these quantile values and each bins have different widths but the same number of transcripts. Note that 100 bins are generated by default and the number of bins can be adjusted manually.
3. Place all the transcripts in the bin to which their mean intensities belong.
4. Label the transcript as an outlier if the difference between classes is larger than the threshold (Default value is 1.2, please see the S1 File and S1 Fig).
5. Remove the outliers labeled in step IV and, then, evaluate the variance of *M* for each bin,
  $${\hat{\sigma }}_{M_{\left(k\right)}}^{2}=\sum_{\forall i_{\left(k\right)}}\frac{{\left(M_{i_{\left(k\right)}}-{\bar{M}}_{\left(k\right)}\right)}^{2}}{n_{\left(k\right)}-1},$$ (3)
  where *i*_(*k*)_, *n*_(*k*)_ and ${\hat{\sigma }}_{M_{\left(k\right)}}^{2}$ denote the genes, the total number of genes, and the variance of *M* on the *k*_*th*_ bin, respectively.
6. Generate the LPE curve by fitting a cubic smoothing spline to the variance along with the bins. This makes the LPE as a function of transcript abundance level.
7. Use the LPE function drawn in step VI to estimate the transcript-specific variance by plugging in the value of each transcript.

Once the variance in each experimental condition is derived, testing a hypothesis on differential expression is similarly conducted as for the analysis with replicates. Note that it is difficult to find the optimal number of bins which might affect the result of the DE test. However, the number of DEGs does not vary much with varying number of bins (S2 Fig). In addition to equal frequency interval binning (using percentiles), we also tried equal spaced interval binning. Although the results did not differ much, the equal spaced interval seemed to be very sensitive to the size of intervals (data not shown).

### Real RNA-Seq data sets

Four real datasets, which had been preprocessed and distributed by *Recount*, were examined [13]. We summarized the characteristics of each dataset below.

1. Sultan’s dataset (two replicates in each condition): Sultan *et al*. [14] performed RNA-Seq experiments in human embryonic kidney and B cell line. With each sample, two replicates were generated, and we analyzed the DE between the two conditions. The dataset provides a case in which a small number of replicates are available.
2. Hammer’s dataset (single replicate in each condition): Hammer *et al*. [15] studied rats with chronic neuropathic pain induced by spinal nerve ligation (SNL) in serial experiments using RNA-Seq. The RNA of rats with SNL for 2 weeks and 2 months was sequenced and compared with that of controls. We performed DE analysis between the SNL and control groups for 2 weeks. The dataset provides a case in which only single replicates are available in each condition.
3. MAQC dataset (7 replicates in each condition): Bullard *et al*. [16] considered two types of biological samples: Ambion’s human brain reference RNA and Stratagene’s human universal reference RNA, referred to as Brain and UHR, respectively. Since the dataset has seven replicates for each condition, the dataset provides a situation in which a large number of replicates are available. Also, the dataset was used to measure how much the result with 7 replicates is reproduced by the analysis with different numbers of replicates.
4. Montgomery’s dataset (60 normal samples): Montgomery *et al*. [17] generated RNA-seq data with 60 unrelated normal Caucasian individuals. Among these 60, 33 individuals are female and 27 individuals are male. The dataset provides an analysis of a large biological samples with unequal group sizes.

### Simulation study

#### Parameter estimation from a real dataset

The purpose of the simulation study was to investigate the ability of DE detection under varying effects of four different factors: effect size (counts difference), DE portion (number), dispersion, and number of replicates in each class. To generate the simulation datasets, we adopted the same assumption that has been made in many other studies [4, 5, 7, 18, 19], i.e., the abundance of transcript *i*, denoted by *X*_*i*_, follows negative binomial distribution, *NB*(*μ*_*i*_,*ϕ*_*i*_). Here, *μ*_*i*_ and *ϕ*_*i*_ are the mean and dispersion parameters, respectively.

We estimated (*μ*_*i*_,*ϕ*_*i*_) from the Montgomery *et al*.’s real data set, one of the largest sample datasets including 60 unrelated normal Caucasian individuals [17]. The total number of transcripts of this data is 52580 and the number of sample, denoted as *N*, is 60. The log-likelihood function for 60 independent observations for each transcript *i* from a negative binomial distribution, given the counts *x*_*i*1_,⋯,*x*_*iN*_, is

$$\begin{array}{l} l\left(\mu_{i},\phi_{i}|x_{i1},\cdots ,x_{iN}\right)\\ =\sum_{j=1}^{N}\log\Gamma \left(x_{ij}+\frac{1}{\phi_{i}}\right)-N\log\Gamma \left(\frac{1}{\phi_{i}}\right)-\sum_{j=1}^{N}\log\Gamma \left(x_{ij}+1\right)-\sum_{j=1}^{N}x_{ij}\log\left(\frac{\mu_{i}\phi_{i}}{1+\mu_{i}\phi_{i}}\right)-\frac{N}{\phi_{i}}\log\left(1+\mu_{i}\phi_{i}\right) \end{array}$$,

a similar form in the supplementary material of the Soneson et al’s paper [7]. The maximum likelihood estimate (MLE) of *μ*_*i*_ was first obtained for each transcript across 60 samples; then, *ϕ*_*i*_ was estimated by numerically maximizing the log-likelihood function using the simulated annealing algorithm [20].

#### Simulated dataset generation

Based on these estimates, we generated simulation datasets as follows. First, we generated 20,000 transcripts in total. Among them 70% were generated from the zero-inflated Poisson distribution with mean 1.25 and zero probability of 0.9. Then, *μ*_*i*_ for the remaining 30% were randomly selected from the values estimated from the real data. Then, for each transcript *i*, we generated transcript counts, *x*_*ij*_ and *y*_*ij*_ from *NB*(*μ*_*i*_,*ϕ*_*i*_ = *ϕ*) in each class. For DE transcripts, we randomly selected *κ*% of the total transcripts and added the effect size *δ* in one of the two classes. In our simulation scenario, we varied the value of *κ*, *δ*, *ϕ*, and the number of replicates per each class as stated below.

- Case I. Different effect size: (500, 1000, and 5000): We varied the effect size ranging from 500 to 5000 in counts, which roughly corresponded to the range of mean and maximum count difference between the two classes in the real dataset with total read counts similar to those of the simulated data (data not shown).
- Case II. Different number of DE transcripts: (1000 and 2000): The number of DE transcripts can vary due to the biological phenomena of interest and can affect the DE detection performance of the tests. We set 5% and 10% of total transcripts (1000 and 2000 transcripts, respectively) as to be DE and observed their effects on the DE detection ability.
- Case III. Different dispersion: (0.01, 0.25, and 0.4): We used values in the interquantile range [0.2, 0.41] of the estimates for the dispersion parameter *ϕ*. We also included 0.01 (Poisson-like behavior) and 0.1 (around minimum of the estimates) values to observe the performance of the methods with small dispersion.
- Case IV. Different number of replicates per each class: (1, 2, 3, and 5 replicates): We repeated the analyses of case I–III with different numbers of replicates per each class, focusing on small sample analysis.

All the cases were repeated 100 times.

### The R package of LPEseq

We implemented the method as a package for the R environment and named it “LPEseq.” It is freely downloadable from our website (http://bibs.snu.ac.kr/software/LPEseq) or Bioconductor. LPEseq uses any kind of count table format as the input file. The package was designed to perform the analysis with only a single command, allowing researchers who are unfamiliar with the R language to use it easily. All the analyses in this work were performed with the package LPEseq. In addition, the whole analysis code used in this work and the LPEseq manual can be found in our website.

### Comparison with other methods

We compared LPEseq with the pre-existing methods edgeR [4, 21], DESeq [5], DESeq2 [11], and NBPSeq [6] with respect to true positive rate (TPR) and FDR. In case of the studies with one sample per each class, DESeq2 and NBPseq were excluded for comparison because they are not applicable. The Benjamini–Hochberg procedure was used to adjust multiple testing problem [22]. Transcripts were reported as DE at an adjusted p-value threshold of 0.05. We compared TPR and FDR defined as the number of true DE transcripts detected divided by the total number of true DE transcripts and the number of false DE transcripts detected divided by the number of DE transcripts detected, respectively. In case of replicated data analysis, we ran the programs using the setting provided in the supplementary material of a review article [7] and the reproducible code provided in the DESeq2 [11]. For the non-replicated data analysis, we used the options recommended by manuals of edgeR and DESeq.

### Gene-set analysis

We used DAVID bioinformatics database [23, 24] for gene set analysis. We uploaded a list of DEGs and performed analysis with gene sets of Gene Ontology terms, KEGG pathways, and Chromosomes. Significantly enriched gene sets were listed based on FDR < 10%.

## Results and Discussion

### Analysis of the simulation data

We investigated the performance of LPEseq regarding the true DE detection ahead of false discovery under 72 combinatorial cases of four different parameters, *δ*, *ϕ*, *κ*, and *m* (i.e., effect size, dispersion, DE proportion, number of replicates, respectively).

The primary purpose of developing LPEseq was to apply it to data with non-replicated samples in each class. In such a case, among the methods under consideration, edgeR and DESeq are applicable. Thus, we compared the results obtained from LPEseq, edgeR and DESeq. The overall performances of the three methods with one replicate in each class are reported in Table 1. In the majority of cases, DESeq produced an error message of “parametric dispersion fit error”, suggesting the use of “local” fit option for the dispersion estimation by the package. Using the “local” fit option solved the problem but resulted in a loss of power where none of true DE transcript was detected, thus generating NAs for FDR except a few cases. However, the performance of edgeR and LPEseq was dramatic. In all cases, LPEseq showed less than 5% FDR and more than 92% TPR. Even though edgeR performed superior than LPEseq in aspect of finding true DE transcripts, showing more than 97% TPR, FDR was not well-controlled. The failure of edgeR to correctly find the DE transcripts might lead to suspicious interpretation of the outcomes. By comparing the results in Table 1, it is obvious that a significant improvement was obtained by applying LPEseq to datasets with non-replicated samples.

**Table 1:** Result comparison with non-replicate per each class.

Parameters			Average FDR (%)			Average TPR (%)		
Effect size	Dispersion	# of DEGs	LPEseq	edgeR	DESeq	LPEseq	edgeR	DESeq
500	0.01	1000	1.39 (0.145)	0.01 (0.032)	90.00 (31.623)	95.11 (0.633)	98.00 (0.462)	0.01 (0.032)
		2000	1.12 (0.263)	0.01 (0.034)	NA	94.43 (0.410)	98.32 (0.207)	0.00 (0.016)
	0.25	1000	2.44 (0.267)	25.38 (0.888)	NA	92.28 (0.763)	97.96 (0.403)	0
		2000	1.77 (0.267)	15.87 (0.305)	NA	92.39 (0.651)	98.30 (0.399)	0
	0.40	1000	4.96 (0.641)	35.95 (9.832)	NA	92.13 (1.069)	97.83 (0.419)	0
		2000	3.20 (0.439)	23.06 (0.791)	NA	93.00 (0.784)	97.90 (0.281)	0
1000	0.01	1000	1.25 (0.280)	0.01 (0.032)	0.57 (0.220)	96.22 (0.596)	99.22 (0.290)	90.88 (0.711)
		2000	1.34 (0.142)	0.01 (0.021)	0.50 (0.137)	95.57 (0.529)	99.36 (0.126)	88.63 (3.014)
	0.25	1000	2.58 (0.667)	26.18 (0.806)	NA	93.69 (0.993)	98.81 (0.384)	0.00 (0.032)
		2000	1.70 (0.363)	15.88 (0.721)	NA	93.81 (0.618)	98.99 (0.124)	0.00 (0.016)
	0.40	1000	4.48 (0.509)	36.31 (0.902)	NA	92.97 (0.581)	98.54 (0.341)	0
		2000	3.28 (0.474)	22.57 (0.533)	NA	93.32 (0.715)	98.76 (0.321)	0
5000	0.01	1000	2.10 (0.740)	0.03 (0.048)	NA	98.83 (0.419)	99.94 (0.070)	0
		2000	2.03 (0.455)	0.00 (0.021)	NA	97.79 (0.300)	99.95 (0.044)	0
	0.25	1000	2.47 (0.586)	25.77 (0.701)	NA	96.42 (0.559)	99.81 (0.137)	0
		2000	1.86 (0.388)	15.65 (0.970)	NA	96.19 (0.504)	99.82 (0.131)	0
	0.40	1000	4.56 (0.595)	36.28 (0.904)	NA	95.74 (0.458)	99.69 (0.145)	0
		2000	3.39 (0.289)	22.77 (0.788)	NA	95.92 (0.330)	99.75 (0.107)	0

In this study, we sought to establish a method applicable to a small number of replicates, not just one in each class. Table 2 summarizes the results of five different methods with three replicates in each class. As can be seen, when replicated samples were used, no method is clearly superior under all conditions and all methods gave satisfactory results. It can be observed from the table that the performance of LPEseq, i.e.,<5% FDR and TPR quantitatively similar to that of other methods in all cases, is consistent with that of other methods. However, it should be noted that, in the NBPSeq method, in some cases the FDR was >5%. Note that we have also investigated the situations of different numbers (2, 5, and 10) of replicates and with a larger number (4,000) of DEGs. Similar results as described above were observed with these simulation settings (data not shown).

**Table 2:** Result comparison with 3 replicates per each class.

Parameters			Average FDR (%)					Average TPR (%)				
Effect size sizeaaaaaaaaaaaasize	Dispersion	# of DEG	LPEseq	edgeR	DESeq	DESeq2	NBPSeq	LPEseq	edgeR	DESeq	DESeq2	NBPSeq
500	0.01	1000	2.76 (0.487)	1.85 (0.613)	2.46 (0.819)	0.90 (0.229)	**6.59** (0.409)	97.14 (0.568)	97.61 (0.409)	97.49 (0.428)	99.88 (0.123)	97.96 (0.688)
		2000	2.31 (0.290)	1.85 (0.326)	2.38 (0.359)	0.83 (0.178)	4.36 (0.528)	97.45 (0.365)	97.87 (0.338)	97.68 (0.368)	99.86 (0.032)	98.00 (0.328)
	0.25	1000	2.34 (0.585)	1.58 (0.365)	2.71 (0.541)	2.99 (0.570)	3.61 (0.561)	94.12 (0.696)	94.40 (0.750)	95.00 (0.670)	96.01 (0.635)	94.91 (0.709)
		2000	1.73 (0.286)	2.20 (0.324)	2.82 (0.467)	2.44 (0.390)	4.46 (0.398)	94.41 (0.377)	94.88 (0.284)	95.38 (0.294)	97.40 (0.331)	95.43 (0.285)
	0.40	1000	2.51 (0.672)	1.53 (0.452)	2.56 (0.460)	3.75 (0.439)	**10.16** (13.718)	93.01 (0.443)	93.50 (0.514)	94.31 (0.507)	88.08 (1.438)	94.10 (0.596)
		2000	1.80 (0.285)	2.36 (0.288)	3.11 (0.321)	2.76 (0.433)	4.49 (0.343)	93.59 (1.688)	94.14 (0.636)	94.90 (0.501)	92.55 (0.650)	94.78 (0.513)
1000	0.01	1000	2.83 (0.523)	1.75 (0.460)	2.30 (0.391)	0.79 (0.284)	5.13 (4.137)	98.26 (0.237)	98.63 (0.250)	98.48 (0.336)	99.97 (0.048)	98.68 (0.257)
		2000	2.40 (0.311)	1.94 (0.235)	2.65 (0.253)	0.66 (0.161)	**6.63** (5.549)	98.38 (0.324)	98.78 (0.678)	98.55 (0.280)	99.98 (0.035)	98.81 (0.307)
	0.25	1000	1.83 (0.383)	1.66 (0.311)	2.42 (0.352)	3.10 (0.363)	3.49 (0.658)	95.12 (0.585)	95.43 (0.598)	95.96 (0.599)	97.21 (0.633)	95.87 (0.540)
		2000	1.75 (0.356)	2.11 (0.336)	2.62 (0.390)	2.14 (0.362)	4.17 (0.439)	95.23 (0.386)	95.59 (0.415)	96.17 (0.332)	98.43 (0.212)	96.12 (0.328)
	0.40	1000	2.09 (0.695)	1.53 (0.433)	2.67 (0.567)	3.64 (0.503)	3.80 (0.565)	94.23 (1.169)	94.78 (0.924)	95.44 (0.829)	89.66 (0.867)	95.24 (0.836)
		2000	1.82 (0.375)	2.15 (0.331)	2.83 (0.421)	2.60 (0.369)	4.37 (0.329)	94.95 (0.402)	95.36 (0.362)	96.07 (0.473)	93.91 (0.693)	95.93 (0.439)
5000	0.01	1000	2.85 (0.465)	2.11 (0.434)	2.37 (0.466)	0.66 (0.312)	4.01 (0.427)	99.84 (0.097)	99.89 (0.145)	99.89 (0.120)	100 (0.000)	99.91 (0.110)
		2000	2.53 (0.430)	2.10 (0.306)	2.16 (0.321)	0.63 (0.160)	4.67 (0.542)	99.84 (0.074)	99.92 (0.054)	99.91 (0.072)	100 (0.000)	99.98 (0.026)
	0.25	1000	2.41 (0.445)	1.62 (0.533)	2.67 (0.539)	3.30 (0.371)	3.96 (0.568)	97.66 (0.295)	97.83 (0.406)	98.47 (0.330)	97.57 (0.538)	98.40 (0.283)
		2000	2.30 (0.275)	2.00 (0.296)	2.47 (0.332)	2.35 (0.510)	5.01 (0.544)	98.33 (0.256)	98.36 (0.254)	98.76 (0.261)	99.34 (0.180)	98.95 (0.251)
	0.40	1000	2.26 (0.512)	1.43 (0.262)	2.30 (0.464)	3.77 (0.679)	3.48 (0.612)	97.01 (0.269)	97.06 (0.395)	97.99 (0.238)	89.13 (1.785)	97.84 (0.227)
		2000	2.04 (0.250)	1.98 (0.303)	2.26 (0.255)	2.66 (0.341)	4.76 (0.424)	97.84 (0.443)	97.91 (0.413)	98.57 (0.298)	95.21 (0.652)	98.68 (0.280)

Here, we considered testing between two classes in RNA-Seq experiments assuming that transcripts (or genes) are independent from each other, which might be unlikely in many real datasets. We also have investigated a situation including correlated transcripts with simulated datasets and the results did not differ much in DE analysis (S3 Fig). Since the methods repeat a univariate statistical test for each genes separately, the inclusion of correlated genes shows little effects on the performance of DE analysis.

### Analysis with real data

We used LPEseq to analyze four different RNA-Seq data sets: non-replicates (Hammer’s dataset), small replicates (Sultan’s dataset), large replicates (MAQC dataset), and large normal sample with unequal group sizes (Montgomery’s dataset). The primary purpose of the analysis was to discover DE transcripts and to compare the results obtained using competing methods.

**Fig 2:**
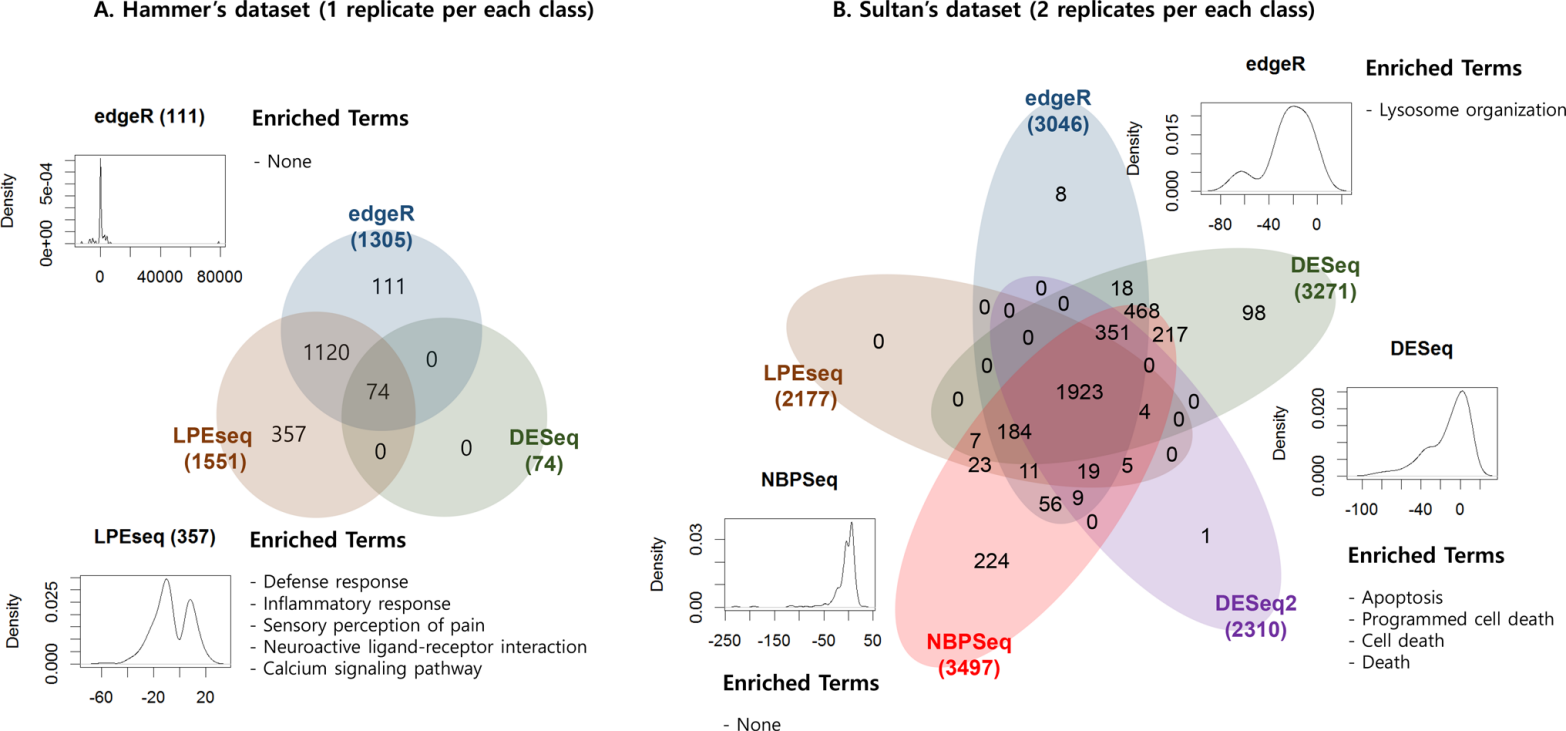
Venn diagrams of differentially expressed (DE) transcripts. Two RNA-Seq datasets with one replicate (A) and two replicates (B) in each class. Five different methods, i.e., LPEseq (brown), edgeR (sky blue), DESeq (green), DESeq2 (violet) and NBPSeq (red) were used. A density plot of the mean difference between classes of uniquely found DE transcripts in each method was indicated. X- and Y-axis represent group mean difference and density. The number in parentheses indicates the total number of DE transcripts found. The criterion used to call DE was Benjamini-Hochberg corrected p-value less than 0.05 for all methods. The enriched terms gene set analysis was performed by DAVID web-tool.

First, we compared the number of DE transcripts found by each method (Fig 2). For the Hammer’s dataset, which has only one sample in each class, LPEseq, edgeR and DESeq were applicable. Hammer *et al*. reported that about 2,000 transcripts were DE and, among them, validated 755 transcripts using quantitative polymerase chain reaction (qPCR) [15]. Interestingly, LPEseq obtained 2,030 DE transcripts, which was similar to what reported by the Hammer *et al*., while edgeR and DESeq detected 1,305 and 74 transcripts as DE, respectively (Fig 2A).

As shown in Fig 2A, 357 genes were additionally called as DEGs by LPEseq. To identify their roles, we performed gene set analysis (GSA). We used gene ontology (GO) terms and KEGG pathways as gene sets and found the terms such as “defence and inflammatory responses”, “sensory perception of pain” and neuroactive ligand-receptor interaction” enriched in higher ranks (< 10% FDR). These terms are strongly related to chronic neuropathic pain induced by SNL, which was the interest of Hammer et al’s paper [15]. GSA with a list of genes only found by edgeR, however, failed to provide relevant information (see also S2 File for a whole list of DEGs and GSA results).

The Sultan’s dataset consists of two different cell types, a kidney and a B-cell, and it would be expected that for such a comparison a large number of genes are differentially expressed. As can been seen in Fig 2B, more DEGs compared to Hammer’s dataset were identified by all methods and many DEGs found by each method were overlapped each other (Fig 2B). The number of DE detected by LPEseq was 2,177, which was the minimum number of DE, while over 3,000 DE were detected by other methods except DESeq2. We noted that the almost DE transcripts found by LPEseq and DESeq2 overlapped with those identified by other methods. However, the other methods included non-overlapped DE transcripts, whose enriched gene functions have no explicit relevance to the comparison of the study (Fig 2B).

To observe characteristics of transcripts uniquely found by each method, we plotted the group mean differences in raw counts using DE transcripts found in each dataset (density plots in Fig 2). As shown in density plot in Fig 2, strong peaks were observed at zero in all competing methods, indicating that there were no considerable mean differences between classes. In fact, these DE transcripts included zero raw counts in both classes, demonstrating that many of these non-overlapping transcripts found were artifacts. For LPEseq, however, the mean difference between classes exhibited a bimodal peak distribution, indicating less likely the DE transcripts found by our proposed method being artifacts.

We also investigated MAQC dataset (comparing brain versus universal reference cell with 7 replicates per each condition) and the similar observations were made. Except edgeR, most methods identified uniquely found by each method and those uniquely identified genes with very small mean difference between conditions showed no enriched term by GSA. Only genes identified by LPEseq were enriched in transcriptional regulation function (See S4 Fig), which is highly activated in brain [25]. By studying real data, it is difficult to be conclusive that LPEseq performs better than other competing methods, but it limits the possibility of producing false calls and the result is robust to the number of replicates in each class (See also S5 and S6 Figs).

To make inferences based upon large as well as small samples through the LPEseq, we analyzed Montgomery’s dataset [17], which consists of 33 normal females and 27 normal males. We first compared the LPE curves evaluated with total samples and two randomly selected samples using the Kolmogorov-Smirnov (KS) test. More specifically, we estimated the variance curve conditioned on the expression levels of the genes using the total samples and we randomly selected one sample from each group and estimate the variance using LPEseq. Then we compared the variance curves by performing a KS test. By repeating 10 times, we obtained 10 p-values from a two-sample KS test and none of them rejected the null hypothesis (S6 Fig), showing the variance estimates from LPEseq method are concordant with the ‘true’ variance estimated from the total samples. We also conducted DE analysis with sex as the group indicator and interpreted the functions of the DEGs using GSA. Since the comparison was made between males and females, it might be reasonable to expect that the genes in sex-chromosomes (X and Y) are more likely to be differentially expressed. As can be seen in the Table 3, genes in Y chromosome were enriched in the list of DEGs identified by each methods, except NBPSeq. Also most of the genes in sex chromosomes were top ranked in the list sorted by increasing p-value (S1 Table). Taken all together, the results show that the LPEseq robustly performs regardless of the sample size and the performance with large sample sizes is comparable to other competing methods.

**Table 3:** Gene set analysis with the chromosome category.

Method	Category	Enriched	Count/Total	%	P-value	Genes	Benjamini
**LPEseq**	Chromosome	Y	5/38	13.88889	5.28e-04	ENSG00000099749, ENSG00000198692, …	0.007371
**edgeR**	Chromosome	19	23/99	25.8427	1.26e-09	ENSG00000129354, ENSG00000105290, …	2.27e-08
	Chromosome	Y	6/99	6.741573	0.002639	ENSG00000099749, ENSG00000198692, …	0.023502
	Chromosome	11	12/99	13.48315	0.009987	ENSG00000175294, ENSG00000172927, …	0.058447
**DESeq**	Chromosome	Y	5/6	83.33333	6.51e-08	ENSG00000099749, ENSG00000198692, …	1.30e-07
**DESeq2**	Chromosome	Y	6/23	27.27273	2.47e-06	ENSG00000099749, ENSG00000198692, …	2.72e-05
**NBPSeq**	Chromosome	12	16/142	11.5942	0.001473	ENSG00000135144, ENSG00000139625, …	0.031907
	Chromosome	19	17/142	12.31884	0.002378	ENSG00000167460, ENSG00000129354, …	0.025846
	Chromosome	11	17/142	12.31884	0.003189	ENSG00000109971, ENSG00000132744, …	0.023149
	Chromosome	17	15/142	10.86957	0.007279	ENSG00000177374, ENSG00000167880, …	0.039383

## Conclusions

We proposed a method for DE analysis with a small number of replicates, especially when a single replicate in each class is available. By extending the LPE method, the proposed method is applicable to the RNA-Seq experiments either with or without replicates per each class. Even though the proposed LPEseq followed the idea of the original LPE method, it is different from that in two aspects: (i) estimating local-pooled variance from different classes assuming them as replicates and (ii) removing outliers derived from the replicates assumption between classes. These two differences make DE analyses with non-replicated datasets feasible. By adding an auxiliary step, our proposed method performed more robustly compared to existing methods regardless of the number of replicates in each class, even when only one replicate in each class was available.

In general, the performance of DE test for RNA-Seq data can be influenced by sequencing depth and the number of replications. By increasing the sequencing depth, it is expected that the accuracy of expression counts will increase. With higher accuracy, we expect that LPEseq could better estimate the variances, resulting in improved performance. However, it was reported that the number of sample replicates is a more significant factor in accurate identification of DE genes in comparison to the sequencing depth [9, 26]. A further detailed investigation of the effect of sequencing depth on the performance of DE test is desirable.

We considered the reproducibility score which represents how much the result made with replicated data is reproduced by the analysis with non-replicated or a smaller number of replicated data in our model development. Using this score, the threshold value for detecting outliers was suggested. By comparing the reproducibility of five methods, LPEseq showed the largest number of DEGs and the highest overlaps between the analyses with subset of samples and total samples, indicating the robust reproducibility regardless of the number of samples (S4 and S5 Figs).

It is worth noting that, even though LPEseq takes the reproducibility into account to the statistical testing without replicates, the scope of any conclusions drawn from it may be limited and need to be interpreted with extra cautions. For example, the outliers flagged in LPEseq may not really be outliers. They can be treated as outliers due to the mean difference between two groups, and this might affect the DE result. We have investigated this effect with MAQC dataset as follows. For MAQC data, there are 7 replicates in each condition. We analyzed all possible combinations of choosing 2 replicates from 7 in the same condition. We performed DE tests as if the two replicates had been obtained from different conditions. The average number of DE found by LPEseq was 5.095 (SD was 5.991) from 52580 transcripts. This analysis shows that the LPEseq method controls the false discovery rate when there are no outliers. Through the analysis with non-replicated data, we believe that the proposed method can be useful to obtain results that are comparable with those obtained from data containing replicates.

Note that the log-transformed count data such as negative binomial data would be an approximation to normal distributed data [27]. Although our LPEseq method treats the log-transformed count data as continuous ones, we believe that the LPE method taking into account the discrete random variables from the NB distribution would have advantage. This extension will be our next research topic.

The proposed idea can be easily extended to more than two comparison problems. For example, more complicated models such as analysis of variance (ANOVA), the “local-pooled-error” and replicates assumption between classes can be applied to the ANOVA problems, with the exceptions that the residual mean sum of squares and residual degrees of freedom are evaluated using observations without outliers in a local bin. The method can also be applicable to test differential expression in the context of high-throughput experiments such as measuring protein expression or DNA methylation.

## Supporting Information

S1 Fig Suggestive threshold value D for non-replicated data analysis. Six different datasets were used to suggest optimal threshold value used in LPEseq method. Reproducibility score versus D values is plotted in blue line with 95% confidence interval colored in red. The D value giving the highest reproducibility score is shown in the center of each plot. The key characteristics of the data appear below each plot.
(TIF)

S2 Fig The number of DEGs with varying number of bins. The number of DEGs is plotted with different number of bins (from 50 to 150 bins).
(TIF)

S3 Fig FDR and TPR of LPEseq with correlated genes. The effect of correlated genes in DE analysis with LPEseq is shown in boxplot for FDR (left) and TPR (right). The different proportions of correlated genes (blue) and the difference correlation coefficient between correlated genes (pink) were denoted in each plot. The analysis was repeated 100 times.
(TIF)

S4 Fig MAQC data analysis. Venn diagram of DEGs is shown for MAQC dataset. Five different methods, i.e., LPEseq (brown), edgeR (sky blue), DESeq (green), DESeq2 (violet) and NBPSeq (red) were used. A density plot of the mean difference between classes of uniquely found DE transcripts in each method was indicated. X- and Y-axis represent group mean difference and density. The number in parentheses indicates the total number of DE transcripts found. The criterion used to call DE was Benjamini-Hochberg corrected p-value less than 0.05 for all methods. The enriched terms gene set analysis was performed by DAVID web-tool.
(TIF)

S5 Fig Reproducibility of the different methods with varying number of samples. The overlapped proportion of DEGs with varying number of technical replicates (left) and biological replicates (right) are shown. The overlap proportion indicates the number of DEGs identified both with subset of samples and with total samples divided by the number of DEGs identified with total samples.
(TIF)

S6 Fig Variance curve comparison. The plot shows the variance curve estimated with different numbers of samples. The X-axis represents log-transformed intensity and the Y-axis does the variance estimates. The solid blue line indicates the ‘true’ variance curve (estimated using the total samples) and all other dashed lines the variance curve estimates using different numbers of samples. None of p-values by a two-sample KS test using the solid blue line and the dashed grey lines were less than 0.05.
(TIF)

S1 Table The most significant DEGs and their chromosome position (top 8 genes are shown). (DOCX)

S1 File Supplementary Note. A file describing how optimal threshold value ‘D’ was defined and evaluated for LPEseq.
(DOCX)

S2 File An excel file containing DEG list and GSEA result of each method for Hammer’s dataset. (XLSX)
