# Supplementary figures and images for: LPEseq: Local-Pooled-Error Test for RNA Sequencing Experiments with a Small Number of Replicates
Source: PLoS One. 2016 Aug 17;11(8):e0159182. doi: 10.1371/journal.pone.0159182 (PMC4988759; doi:10.1371/journal.pone.0159182)

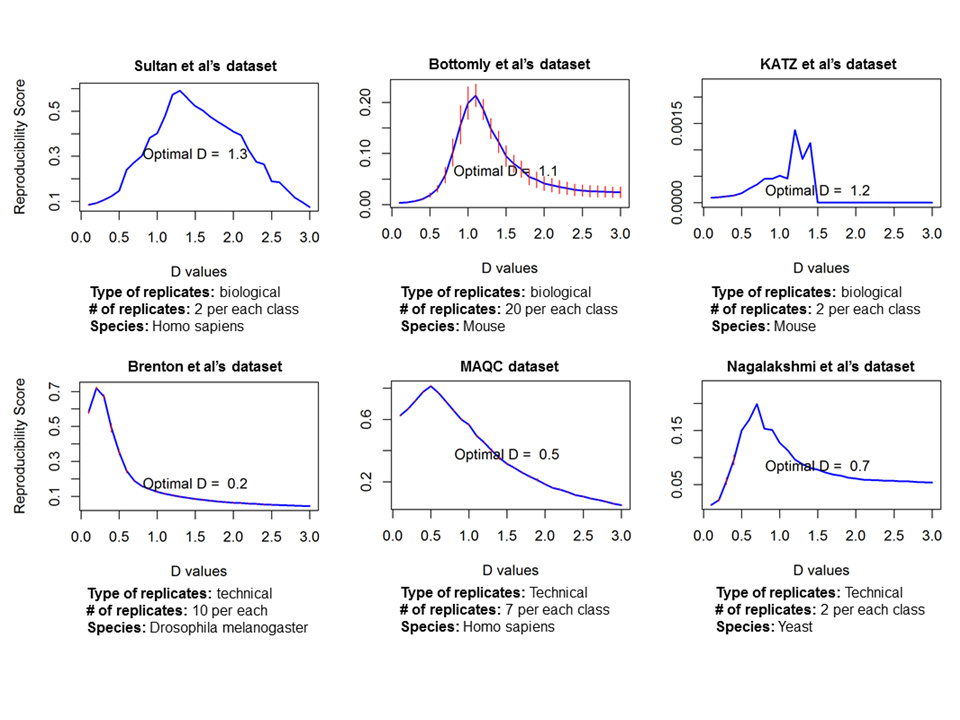

Supplement: S1 Fig — Six different datasets were used to suggest optimal threshold value used in LPEseq method. Reproducibility score versus D values is plotted in blue line with 95% confidence interval colored in red. The D value giving the highest reproducibility score is shown in the center of each plot. The key characteristics of the data appear below each plot. (TIF) [file pone.0159182.s001.TIF]

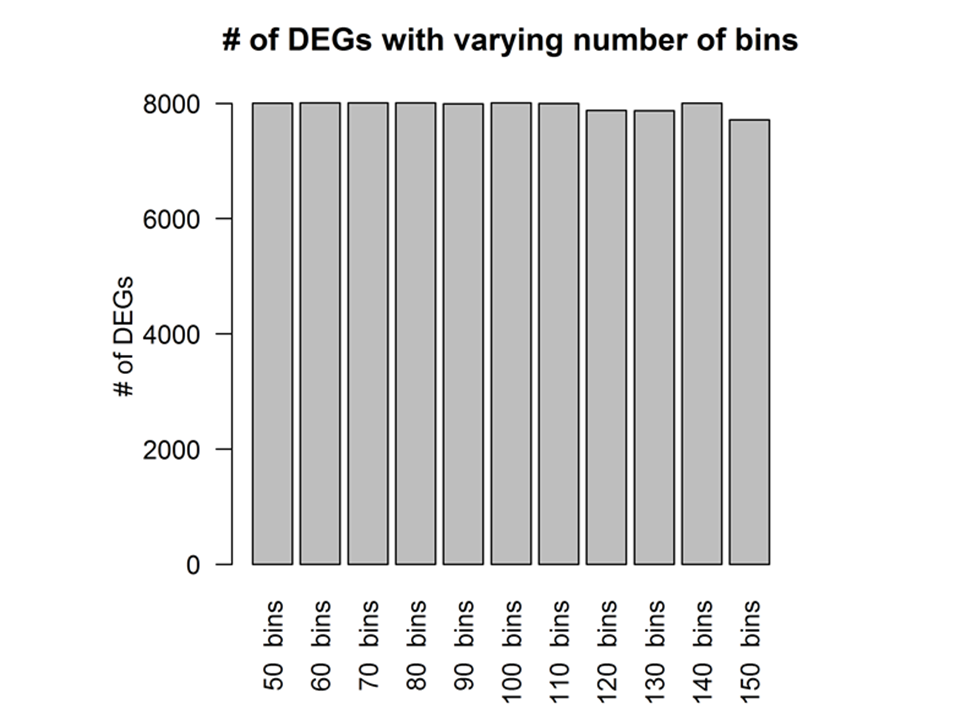

Supplement: S2 Fig — The number of DEGs is plotted with different number of bins (from 50 to 150 bins). (TIF) [file pone.0159182.s002.TIF]

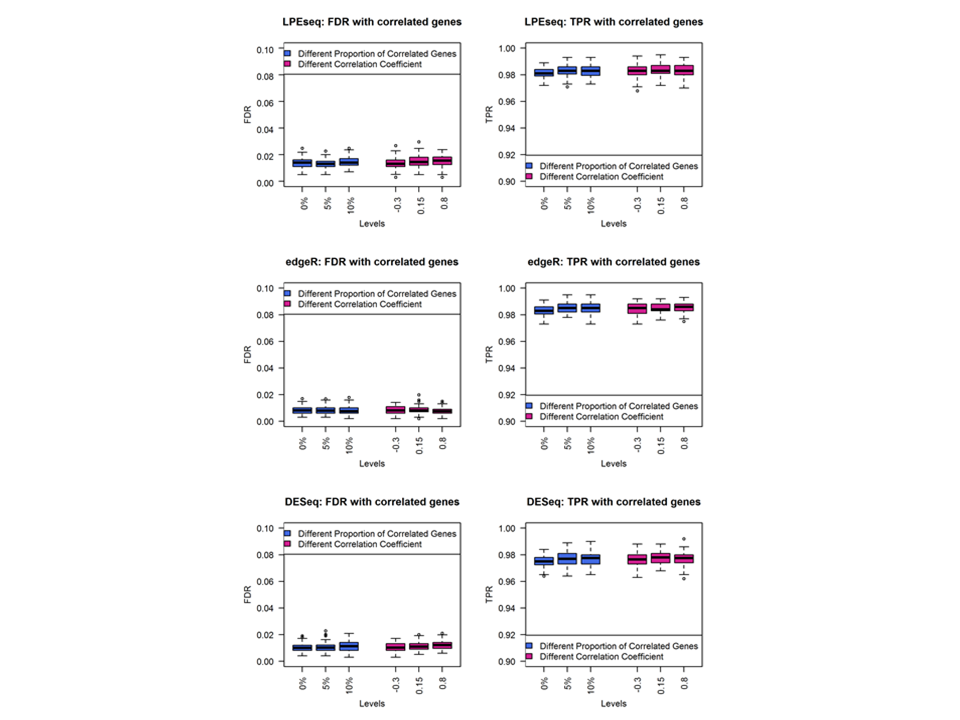

Supplement: S3 Fig — The effect of correlated genes in DE analysis with LPEseq is shown in boxplot for FDR (left) and TPR (right). The different proportions of correlated genes (blue) and the difference correlation coefficient between correlated genes (pink) were denoted in each plot. The analysis was repeated 100 times. (TIF) [file pone.0159182.s003.TIF]

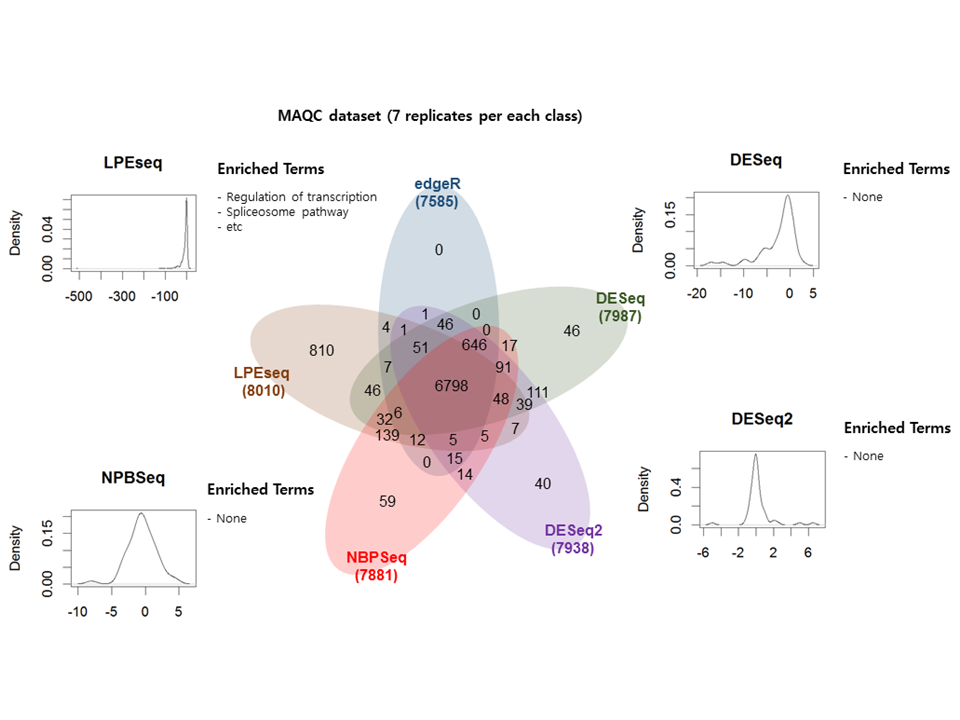

Supplement: S4 Fig — Venn diagram of DEGs is shown for MAQC dataset. Five different methods, i.e., LPEseq (brown), edgeR (sky blue), DESeq (green), DESeq2 (violet) and NBPSeq (red) were used. A density plot of the mean difference between classes of uniquely found DE transcripts in each method was indicated. X- and Y-axis represent group mean difference and density. The number in parentheses indicates the total number of DE transcripts found. The criterion used to call DE was Benjamini-Hochberg corrected p-value less than 0.05 for all methods. The enriched terms gene set analysis was performed by DAVID web-tool. (TIF) [file pone.0159182.s004.TIF]

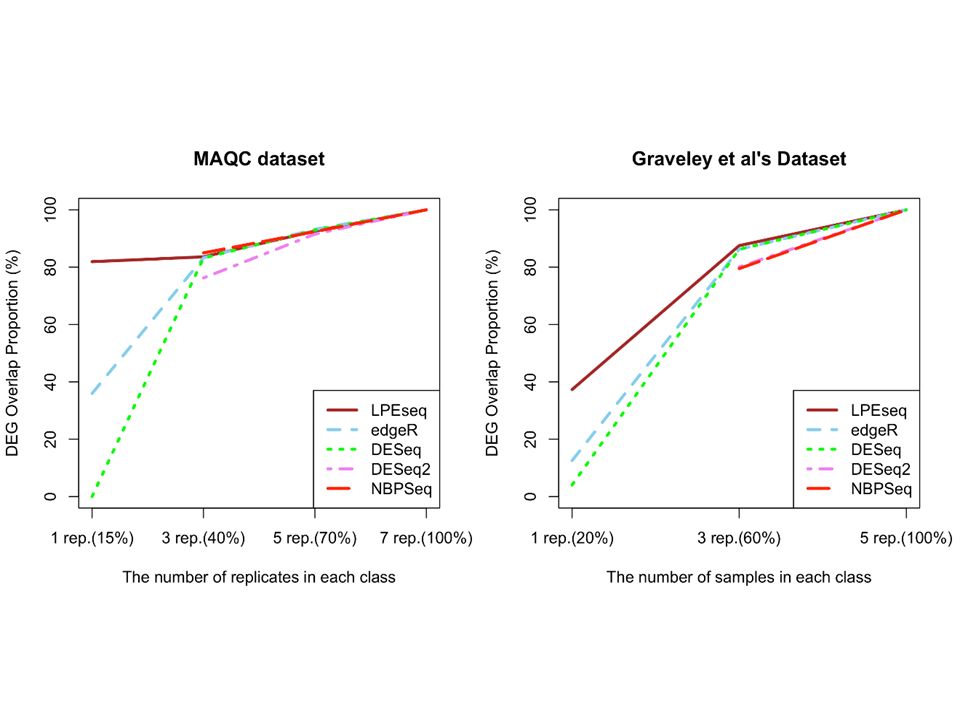

Supplement: S5 Fig — The overlapped proportion of DEGs with varying number of technical replicates (left) and biological replicates (right) are shown. The overlap proportion indicates the number of DEGs identified both with subset of samples and with total samples divided by the number of DEGs identified with total samples. (TIF) [file pone.0159182.s005.TIF]

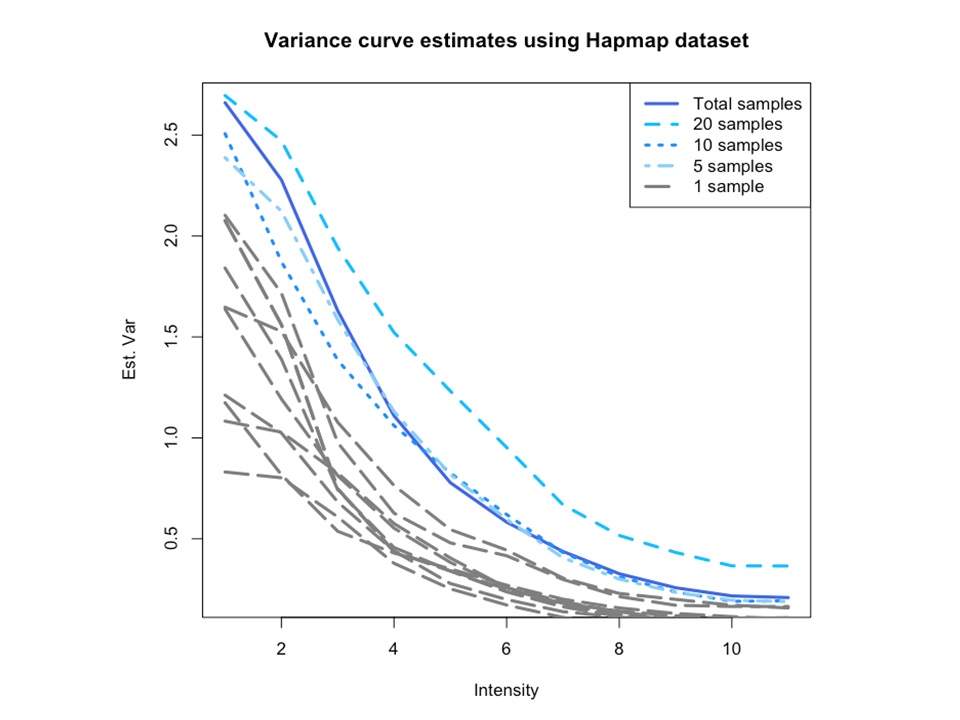

Supplement: S6 Fig — The plot shows the variance curve estimated with different numbers of samples. The X-axis represents log-transformed intensity and the Y-axis does the variance estimates. The solid blue line indicates the ‘true’ variance curve (estimated using the total samples) and all other dashed lines the variance curve estimates using different numbers of samples. None of p-values by a two-sample KS test using the solid blue line and the dashed grey lines were less than 0.05. (TIF) [file pone.0159182.s006.TIF]
