# Supplementary material for: LPEseq: Local-Pooled-Error Test for RNA Sequencing Experiments with a Small Number of Replicates
Source: PLoS One. 2016 Aug 17;11(8):e0159182. doi: 10.1371/journal.pone.0159182 (PMC4988759; doi:10.1371/journal.pone.0159182)
Supplement: S1 Table — (DOCX) [file pone.0159182.s007.docx]

**S1 Table.** The most significant DEGs and their chromosome position (top 8 genes are shown)

| Method (total # of DEGs) | ID | | Chromosome | Adj. P-value (O(e-10)) | |
| --- | --- | --- | --- | --- | --- |
| LPEseq (38) | ENSG00000022556 | | 19 | 0 | |
|  | ENSG00000099749 | | Y | 0 | |
|  | ENSG00000129824 | | Y | 0 | |
|  | ENSG00000154620 | | Y | 0 | |
|  | ENSG00000157828 | | Y | 3.49e-08 | |
|  | ENSG00000076716 | | X | 2.734e-07 | |
|  | ENSG00000198692 | | Y | 2.734e-07 | |
|  | ENSG00000102962 | | 16 | e.6378e-06 | |
|  | **…** |  | | |  |
| edgeR (99) | ENSG00000099749 | | Y | 0 | |
|  | ENSG00000154620 | | Y | 0 | |
|  | ENSG00000157828 | | Y | 0 | |
|  | ENSG00000198692 | | Y | 0 | |
|  | ENSG00000006757 | | X | 1.8e-09 | |
|  | ENSG00000129824 | | Y | 2.39e-08 | |
|  | ENSG00000183878 | | Y | 4.3871e-06 | |
|  | ENSG00000174938 | | 16 | 1.17975e-05 | |
|  | **…** |  | | |  |
| DESeq (6) | ENSG00000129824 | | Y | 0 | |
|  | ENSG00000099749 | | Y | 0 | |
|  | ENSG00000154620 | | Y | 0 | |
|  | ENSG00000157828 | | Y | 2.3e-09 | |
|  | ENSG00000198692 | | Y | 6.52e-08 | |
|  | ENSG00000006757 | | X | 0.0001499369 | |
| DESeq2 (23) | ENSG00000154620 | | Y | 0 | |
|  | ENSG00000099749 | | Y | 0 | |
|  | ENSG00000157828 | | Y | 0 | |
|  | ENSG00000006757 | | X | 0 | |
|  | ENSG00000198692 | | Y | 1e-10 | |
|  | ENSG00000174938 | | 16 | 0.0003820809 | |
|  | ENSG00000183878 | | Y | 0.001595929 | |
|  | ENSG00000205890 | | 16 | 0.0023876896 | |
|  | **…** |  | | |  |
| NBPSeq (142) | ENSG00000129824 | | Y | 0 | |
|  | ENSG00000137573 | | 8 | 0 | |
|  | ENSG00000101210 | | 20 | 0 | |
|  | ENSG00000165949 | | 14 | 0 | |
|  | ENSG00000099749 | | Y | 0 | |
|  | ENSG00000154620 | | Y | 0 | |
|  | ENSG00000176165 | | 14 | 0 | |
|  | ENSG00000138755 | | 4 | 0 | |
|  | **…** |  | | |  |
