# Supplementary material for: LPEseq: Local-Pooled-Error Test for RNA Sequencing Experiments with a Small Number of Replicates
Source: PLoS One. 2016 Aug 17;11(8):e0159182. doi: 10.1371/journal.pone.0159182 (PMC4988759; doi:10.1371/journal.pone.0159182)
Supplement: S1 File — A file describing how optimal threshold value ‘D’ was defined and evaluated for LPEseq. (DOCX) [file pone.0159182.s008.docx]

**[S1 File]**

Supplementary materials section for

“LPEseq: Local-pooled-error test for RNASequencing experiments with a small number of replicates”

Jungsoo Gim^1^, Sungho Won^2^, and Taesung Park^3*^

^1^Institute of Health and Environment, Seoul National University, 1 Gwanak-ro, Gwanak-gu, Seoul, Korea

^2^Graduate School of Public Health, Seoul National University, 1 Gwanak-ro, Gwanak-gu, Seoul, Korea

^3^Department of Statistics, Seoul National University, 1 Gwanak-ro, Gwanak-gu, Seoul, Korea

^*^To whom correspondence should be addressed.

Email: tspark@stats.snu.ac.kr

# Supplementary Note

## Defining Reproducibility score

To perform DE test without replicates (one sample per each class), LPEseq assumes that a majority of transcripts in two different classes (e.g., disease and control) is not affected by the different condition, and thus remains the same. By this assumption, LPEseq first identifies the transcripts whose expression is largely different between classes, and flags them as possible outliers. Then removed outliers, LPE variance between classes is evaluated in the same way as was done for the analysis with replicates. For outlier detection, both statistical tests (i.e., using Cook’s distance and Z-score) and labeling methods (i.e., fold change) can be applied. To statistically test whether a certain data is an outlier, a distribution that the data follows should be assumed. Unlike statistical test-based methods, however, labeling methods classify data either as normal observations or outlying observations according to a specific threshold. Since different methods will produce different results, various outlier detection methods are implemented in LPEseq R-package. Here we described how we suggested the threshold value for the labeling method used in LPEseq (for the analysis without replicates).

The reproducibility score represents how much the result made with replicated data is reproduced by the analysis without replicates. For this score, two criteria were used: The difference of the number of DE transcripts found by the analyses with and without replicates and the overlaps between them. To be more specific, the number of DE transcripts found with non-replicated data ($n_{no.rep}$), that with replicated data ($n_{rep}$), and the number of the overlapped transcripts between $n_{no.rep}$ and $n_{rep}$ ($n_{overlap}$) defines the reproducibility score, *i.e.,*

$$Rep.Score=R_{overlap}\times R_{DE}$$

where$R_{overlap}(=n_{overlap}/n_{no.rep})$and $R_{DE}(=\min(n_{no.rep}/n_{rep},n_{rep}/n_{no.rep})$ indicate the ratio of the overlapped transcripts in the DE transcripts found by the analysis without replicates, and the ratio of the numbers of DE transcripts found by the analyses with and without replicates.

## Interpretation of reproducibility score

It is worth of noting that other types of score can be defined. However, we found that the reproducibility score was easy to interpret the result and useful to provide the suggestive threshold value. To explain the effect of considering two criteria ($R_{DE}$ and $R_{overlap}$) simultaneously, consider the following situation. Suppose we have identified 1000 DE transcripts from the analysis of data with replicates. Data without replicates can be generated by taking one sample per each class and test for DE can be performed with a specific threshold value $D$. If a small $D$ value were used, a large number of DE transcripts are found and many DE transcripts are overlapped to each other. However, the discrepancy between the numbers of DE transcripts makes $R_{DE}$ small.

As the threshold hold value $D$ gets larger, LPEseq finds less DE transcripts (small $n_{no.rep}$) but a comparable number to $n_{rep}$. If the $n_{no.rep}$ becomes close to the $n_{rep}$ and both $R_{DE}$ and $R_{overlap}$ approaches to 1. It is worth of noting that if the DE transcripts found by the analyses with and without replicates are exactly the same, then both $R_{DE}$ and $R_{overlap}$ become 1 and thus $Rep.Score=1$. Above a certain threshold, however, larger $D$ makes $R_{DE}$ smaller because of the discrepancy between $n_{rep}$ and $n_{no.rep}$ while $R_{overlap}$ remains close to 1. In this case, overall $Rep.Score$ gets smaller (Fig. S1).

## Evaluating suggestive threshold D

To suggest an optimal threshold value for an application to DE analysis without replicates, Reproducibility scores with varying threshold values were evaluated in six different real datasets: Sultan et al’s dataset (1), Bottomly et al’s dataset (2), Brenton et al’s dataset (3), MAQC dataset (4) Katz et al’s dataset (5) and Nagalakshmi et al’s dataset (6). Among these six datasets, three have biological replicates and the other three have technical replicates. Brief characteristics of each dataset can be found in Fig. S1.

For each dataset, we performed the DE analysis with replicates and obtained the list of DE transcripts. Then we selected a single sample per each condition and re-performed the analysis with varying specific threshold values. The optimal score was determined by repeating the analysis for all possible combinations of samples with increasing threshold values (from 0.1 to 3.0).

As can be seen in Fig. S1, the average threshold value giving the highest optimality score with biological replicate datasets (technical replicate datasets) was 1.2 (0.5, rounded to the second decimal place). Therefore we recommended this threshold for the DE analysis without replicates using LPEseq. Please note that the suggested value here can be data-specific and might not be the best choice for other datasets. If the variance among samples is expected large, then the higher threshold value of D is recommended (above 2).

# Supporting Figures and Figure Captions


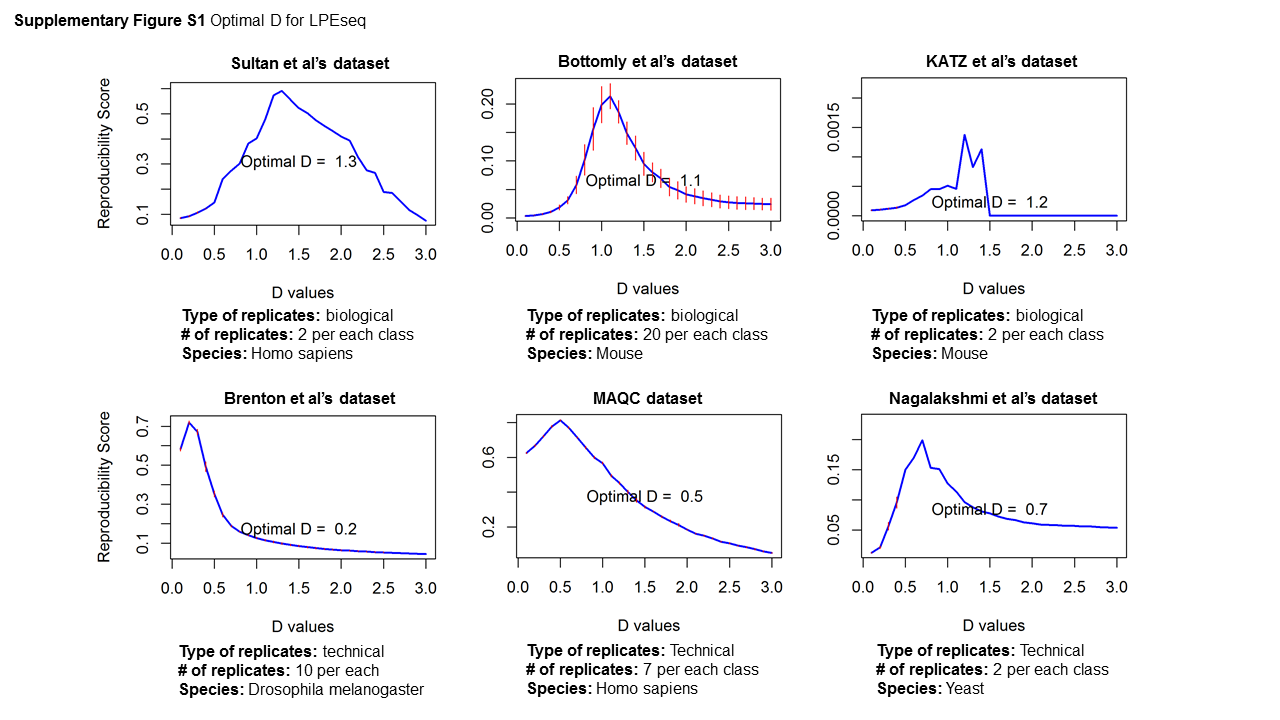


S1 Fig: **Suggestive threshold value D for non-replicated data analysis.** Six different datasets were used to suggest optimal threshold value used in LPEseq method. Reproducibility score versus D values is plotted in blue line with 95% confidence interval colored in red. The D value giving the highest reproducibility score is shown in the center of each plot. The key characteristics of the data appear below each plot.


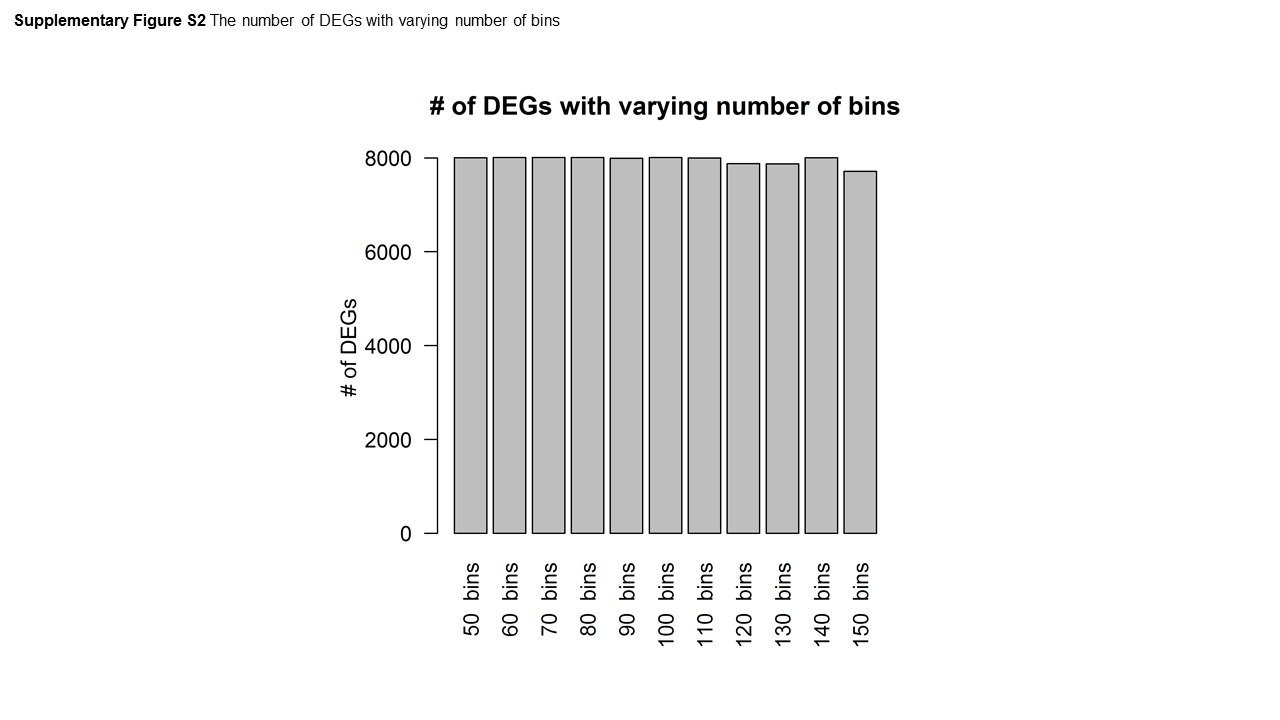


S2 Fig: **The number of DEGs with varying number of bins.** The number of DEGs is plotted with different number of bins (from 50 to 150 bins).


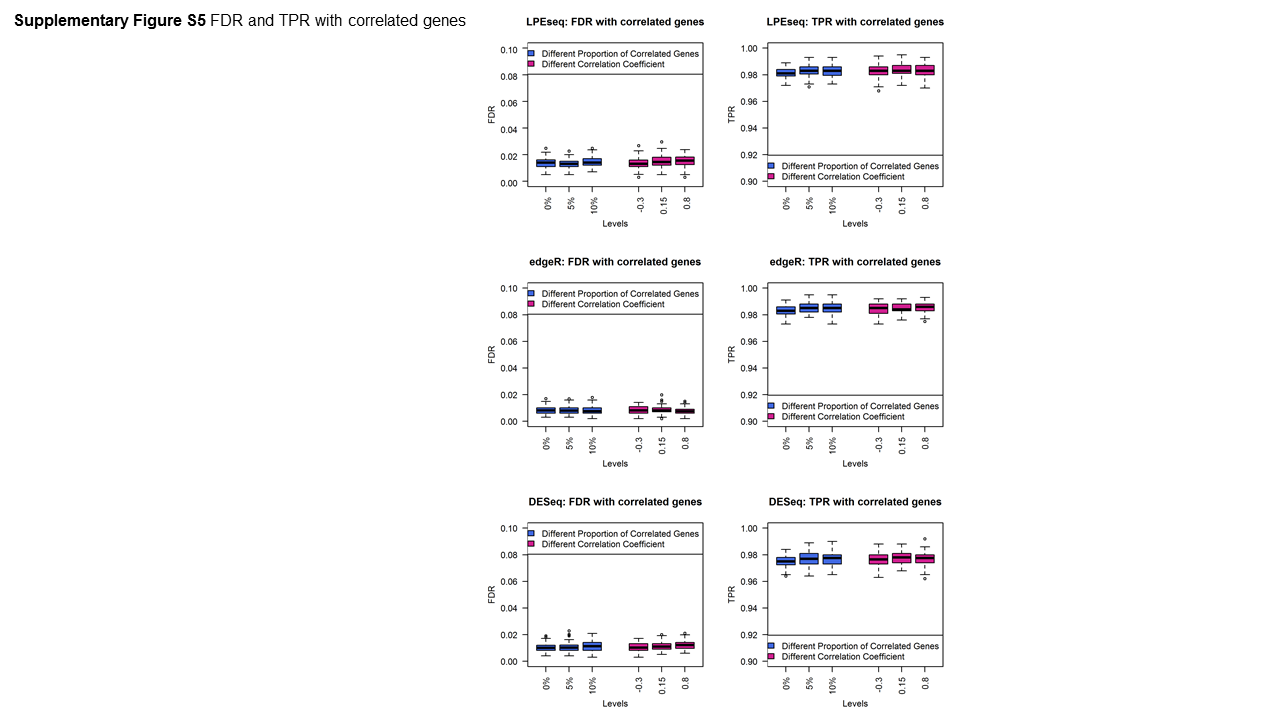


S3 Fig: **FDR and TPR of LPEseq with correlated genes.** The effect of correlated genes in DE analysis with LPEseq is shown in boxplot for FDR (left) and TPR (right). The different proportions of correlated genes (blue) and the difference correlation coefficient between correlated genes (pink) were denoted in each plot. The analysis was repeated 100 times.


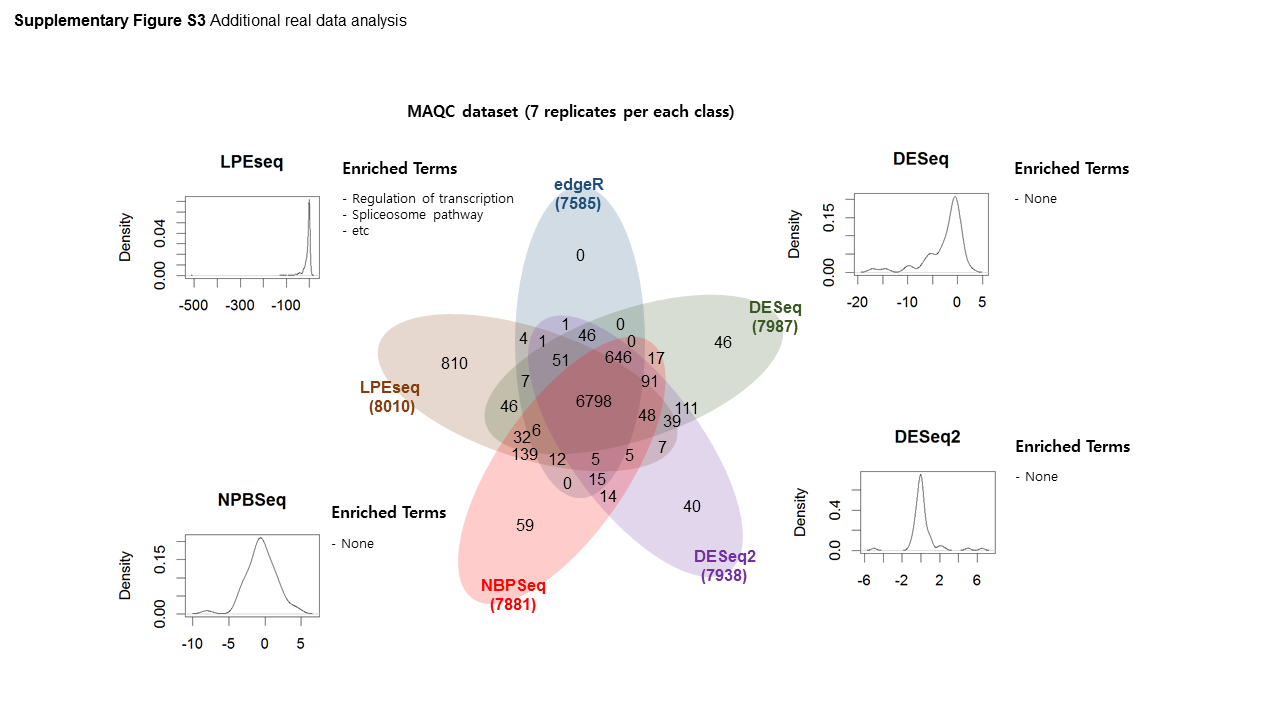


S4 Fig: **MAQC data analysis.** Venn diagram of DEGs is shown for MAQC dataset. Five different methods, i.e., LPEseq (brown), edgeR (sky blue), DESeq (green), DESeq2 (violet) and NBPSeq (red) were used. A density plot of the mean difference between classes of uniquely found DE transcripts in each method was indicated. X- and Y-axis represent group mean difference and density. The number in parentheses indicates the total number of DE transcripts found. The criterion used to call DE was Benjamini-Hochberg corrected p-value less than 0.05 for all methods. The enriched terms gene set analysis was performed by DAVID web-tool.


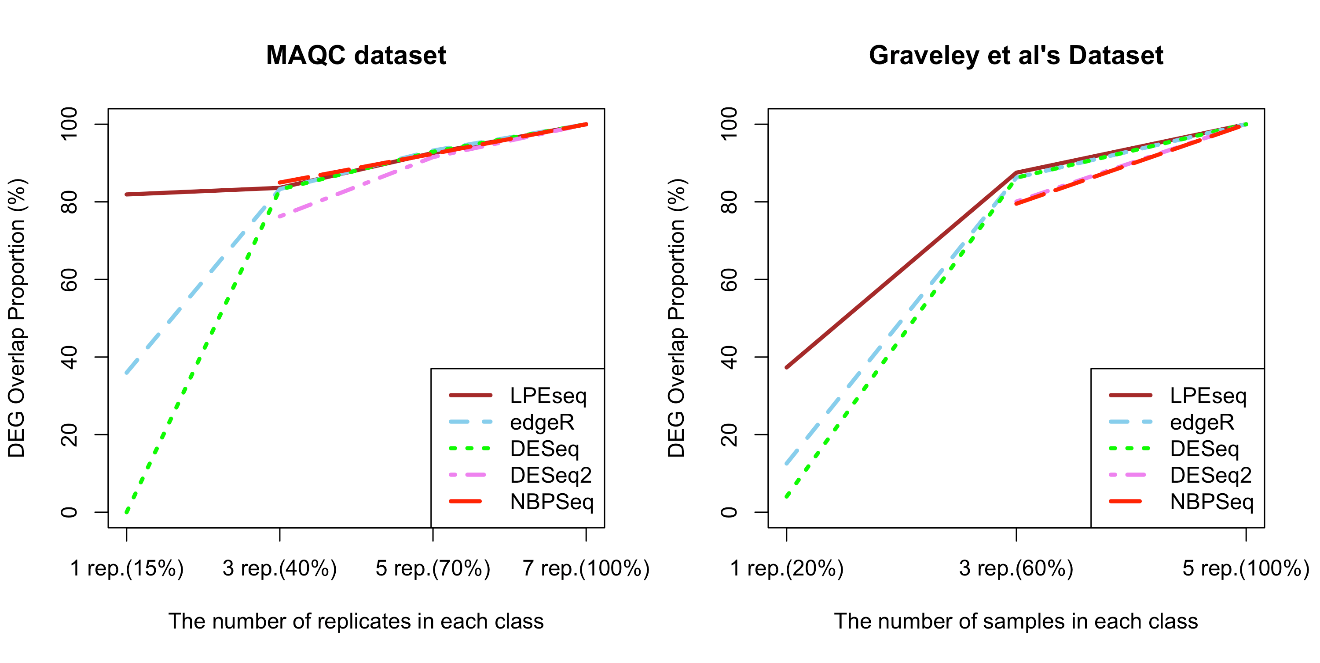


S5 Fig: **Reproducibility of the different methods with varying number of samples.** The overlapped proportion of DEGs with varying number of technical replicates (left) and biological replicates (right) are shown. The overlap proportion indicates the number of DEGs identified both with subset of samples and with total samples divided by the number of DEGs identified with total samples.


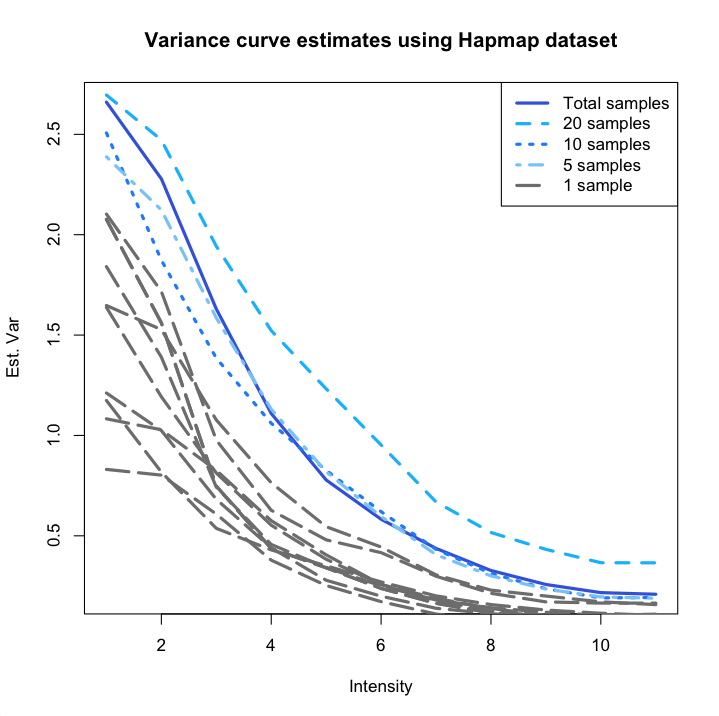


S6 Fig: **Variance curve comparison.** The plot shows the variance curve estimated with different numbers of samples. The X-axis represents log-transformed intensity and the Y-axis does the variance estimates. The solid blue line indicates the ‘true’ variance curve (estimated using the total samples) and all other dashed lines the variance curve estimates using different numbers of samples. None of p-values by a two-sample KS test using the solid blue line and the dashed grey lines were less than 0.05.

S1 Table. The most significant DEGs and their chromosome position (top 8 genes are shown)

| Method (total # of DEGs) | ID | | Chromosome | Adj. P-value (O(e-10)) | |
| --- | --- | --- | --- | --- | --- |
| LPEseq (38) | ENSG00000022556 | | 19 | 0 | |
|  | ENSG00000099749 | | Y | 0 | |
|  | ENSG00000129824 | | Y | 0 | |
|  | ENSG00000154620 | | Y | 0 | |
|  | ENSG00000157828 | | Y | 3.49e-08 | |
|  | ENSG00000076716 | | X | 2.734e-07 | |
|  | ENSG00000198692 | | Y | 2.734e-07 | |
|  | ENSG00000102962 | | 16 | e.6378e-06 | |
|  | **…** |  | | |  |
| edgeR (99) | ENSG00000099749 | | Y | 0 | |
|  | ENSG00000154620 | | Y | 0 | |
|  | ENSG00000157828 | | Y | 0 | |
|  | ENSG00000198692 | | Y | 0 | |
|  | ENSG00000006757 | | X | 1.8e-09 | |
|  | ENSG00000129824 | | Y | 2.39e-08 | |
|  | ENSG00000183878 | | Y | 4.3871e-06 | |
|  | ENSG00000174938 | | 16 | 1.17975e-05 | |
|  | **…** |  | | |  |
| DESeq (6) | ENSG00000129824 | | Y | 0 | |
|  | ENSG00000099749 | | Y | 0 | |
|  | ENSG00000154620 | | Y | 0 | |
|  | ENSG00000157828 | | Y | 2.3e-09 | |
|  | ENSG00000198692 | | Y | 6.52e-08 | |
|  | ENSG00000006757 | | X | 0.0001499369 | |
| DESeq2 (23) | ENSG00000154620 | | Y | 0 | |
|  | ENSG00000099749 | | Y | 0 | |
|  | ENSG00000157828 | | Y | 0 | |
|  | ENSG00000006757 | | X | 0 | |
|  | ENSG00000198692 | | Y | 1e-10 | |
|  | ENSG00000174938 | | 16 | 0.0003820809 | |
|  | ENSG00000183878 | | Y | 0.001595929 | |
|  | ENSG00000205890 | | 16 | 0.0023876896 | |
|  | **…** |  | | |  |
| NBPSeq (142) | ENSG00000129824 | | Y | 0 | |
|  | ENSG00000137573 | | 8 | 0 | |
|  | ENSG00000101210 | | 20 | 0 | |
|  | ENSG00000165949 | | 14 | 0 | |
|  | ENSG00000099749 | | Y | 0 | |
|  | ENSG00000154620 | | Y | 0 | |
|  | ENSG00000176165 | | 14 | 0 | |
|  | ENSG00000138755 | | 4 | 0 | |
|  | **…** |  | | |  |

# References

1. Sultan M, Schulz MH, Richard H, Magen A, Klingenhoff A, Scherf M, et al. A global view of gene activity and alternative splicing by deep sequencing of the human transcriptome. Science. 2008;321(5891):956-60.

2. Bottomly D, Walter NA, Hunter JE, Darakjian P, Kawane S, Buck KJ, et al. Evaluating gene expression in C57BL/6J and DBA/2J mouse striatum using RNA-Seq and microarrays. PloS one. 2011;6(3):e17820.

3. Graveley BR, Brooks AN, Carlson JW, Duff MO, Landolin JM, Yang L, et al. The developmental transcriptome of Drosophila melanogaster. Nature. 2011;471(7339):473-9.

4. Bullard JH, Purdom E, Hansen KD, Dudoit S. Evaluation of statistical methods for normalization and differential expression in mRNA-Seq experiments. BMC bioinformatics. 2010;11:94.

5. Katz Y, Wang ET, Airoldi EM, Burge CB. Analysis and design of RNA sequencing experiments for identifying isoform regulation. Nature methods. 2010;7(12):1009-15.

6. Nagalakshmi U, Wang Z, Waern K, Shou C, Raha D, Gerstein M, et al. The transcriptional landscape of the yeast genome defined by RNA sequencing. Science. 2008;320(5881):1344-9.
